# Ultrasonic shears assistance can shorten the console time in robotic gastrectomy for early gastric cancer

**DOI:** 10.1186/s13104-015-1432-1

**Published:** 2015-09-15

**Authors:** Yoshihiro Kakeji, Daisuke Kuroda, Tetsu Nakamura, Satoshi Suzuki, Masashi Yamamoto, Shingo Kanaji, Tatsuya Imanishi, Kenichi Tanaka

**Affiliations:** Division of Gastrointestinal Surgery, Department of Surgery, Graduate School of Medicine, Kobe University, Kobe, Japan

**Keywords:** Robotic surgery, Gastric cancer, Ultrasonic shears

## Abstract

**Background:**

Robotic gastric surgery has been introduced and is being performed in many Japanese facilities. There are some limitations of devices capable to be used in the robotic arms in the da Vinci Surgical System. We have reviewed our first ten cases with early gastric cancer who underwent robot-assisted gastrectomy and have compared the operative time between cases who underwent the operation only with an electric cautery device and those in whom laparoscopic coagulating shears (LCS) through an assistant port were used.

**Findings:**

We used an electric cautery device only in cases 1–3, and LCS in cases 4–10 except case 9. The mean operative time was 454 min in cases where only robotic devices were used and 414 min in those with LCS assist. The mean console time of 251 min in those with LCS assist was significantly shorter than that of 306 min in cases where only robotic devices were used. The number of dissected lymph nodes was satisfactory, and the estimated blood loss was small. Postoperative complications in two cases were slight and transient with short hospital stay.

**Conclusion:**

Assistant use of ultrasonic shears is useful to shorten the console time in robotic gastrectomy.

## Findings

Gastric cancer is the fifth most prevalent neoplasm worldwide [1], and surgery is the most important curative treatment for this malignancy. Various technical special procedures have been tried, and laparoscopic gastrectomy with lymph node dissection has been shown to be not only feasible but also safe, achieving better early postoperative outcomes when compared to conventional open gastrectomy [2–4]. A minimally invasive approach has gained increasing acceptance due to improved postoperative outcomes. Robotic surgery is an emerging technology that allows laparoscopic procedures to be carried out in many surgical situations, and the da Vinci Surgical System has been introduced with encouraging results [5, 6]. Robotic gastrectomy is a feasible and safe procedure in the hands of experienced laparoscopic surgeons [7, 8]. In Japan, there has been some limitation in the availability of devices in the da Vinci Surgical System. For example, we cannot use ultrasonic shears which is useful for lymph node dissection in the da Vinci Surgical System. In this article, we analyzed our initial experience in robotic gastrectomy with the da Vinci S Surgical System.

## Methods

Use of the da Vinci S Surgical System at Kobe University, Kobe, Japan began in February 2011. The initial ten consecutive patients with early gastric cancer who were preoperatively diagnosed as cT1N0M0 between February 2011 and April 2012 and subsequently underwent robot-assisted gastrectomy (RAG) were used in the analyses. The decision to apply RAG only in patients with early gastric cancer is based on the recommendations of the Japanese treatment guideline for gastric cancer [9] as well as the fact that the oncological safety of minimally invasive surgery for advanced gastric cancer remains controversial [3]. The clinicopathologic characteristics, postoperative outcomes, and postoperative morbidities of each case are shown in Table 1. Before surgery, the details of the procedure was explained to all the patients, and appropriately written informed consent was obtained. This clinical study was approved by the Institutional Review Board of Kobe University Hospital (No. 1110) and registered in the University Hospital Medical Information Network (UMIN) Clinical Trials Registry (UMIN-CTR) (UMIN000004181, registered 10 September 2010).

**Table 1:** Patients’ clinicopathologic characteristics

Case #	Age	Sex	BMI	pT	pN	Blood loss (g)	Operative time (min)	Console time (min)	Postop. hosp. stay	Robotic only or LCS assist
1	61	M	23.5	pT1a	pN0	60	426	275	11	Robotic only
2	71	M	21.3	pT1b	pN0	30	408	304	11	Robotic only
3	57	F	26.0	pT2	pN0	60	462	290	10	Robotic only
4	70	M	23.5	pT1a	pN0	94	386	225	12	LCS assist
5	44	F	18.0	pT1b	pN0	0	347	204	16	LCS assist
6	59	F	21.9	pT1b	pN0	65	404	250	11	LCS assist
7	50	F	21.3	pT1a	pN0	90	441	243	12	LCS assist
8	68	M	26.8	pT1b	pN0	155	461	273	11	LCS assist
9	74	M	21.4	pT1b	pN0	90	518	355	10	Robotic only
10	64	M	24.6	pT1a	pN0	45	444	308	12	LCS assist

**Fig. 1:**
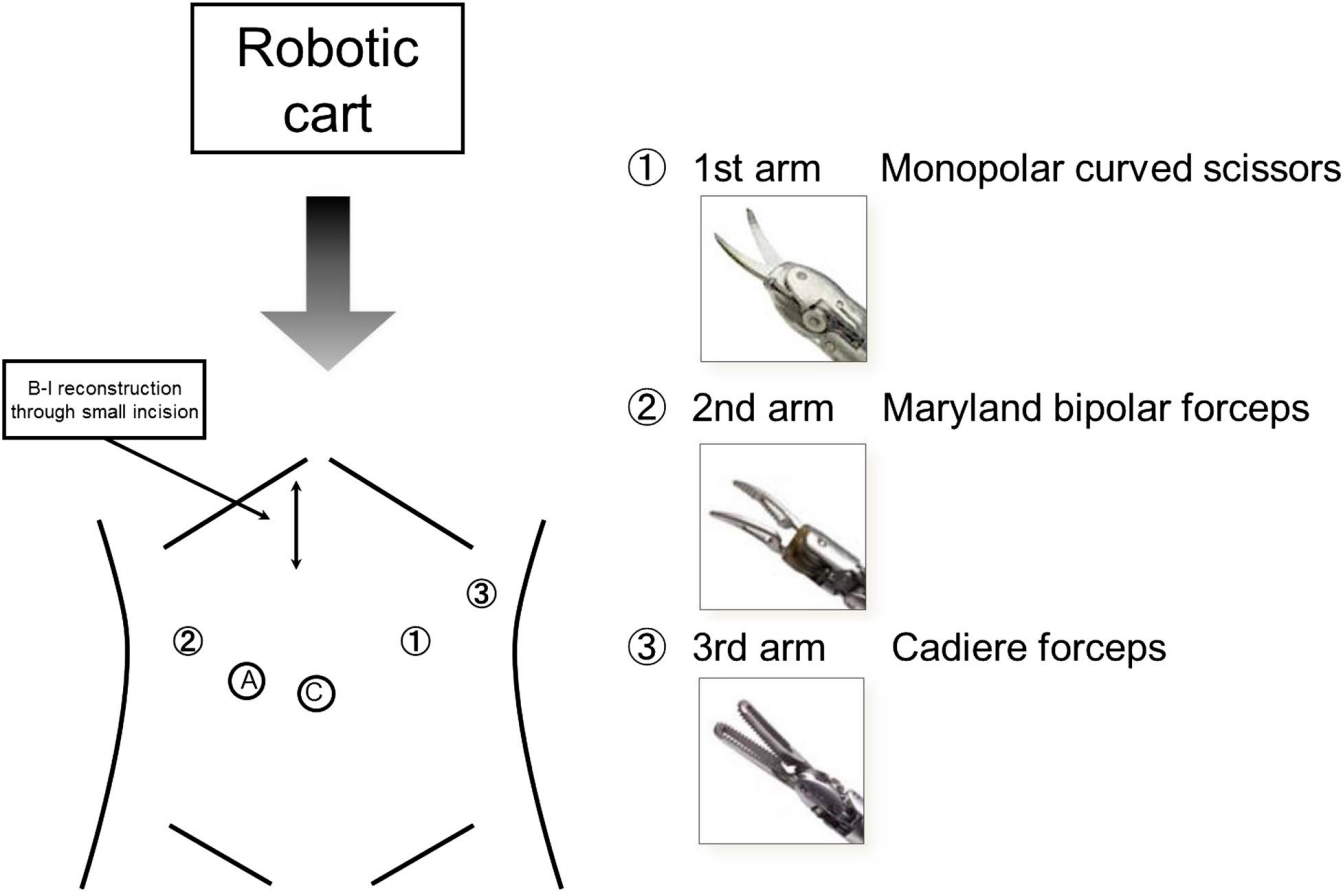
Locations of the ports in robotic gastrectomy. *C* camera port. *A* assist port. ①: 1st robot arm port. ②: 2nd robot arm port. ③: 3rd robot arm port

All the operations in the current study were performed by one surgeon (DK) and the same assistants (TN and SS). The da Vinci S Surgical System was used in all procedures except gastro-duodenal or gastro-jejunal anastomoses. All patients underwent distal gasrectomy with D1+ or D2 lymphadenectomy. Figure 1 shows the locations of the trocars. We used one 12-mm trocar for the camera, three 8-mm trocars for the robot arms, and one 12-mm trocar for the surgical assistant. Almost all of the surgical procedures in the abdominal cavity are identical to those of laparoscopic gastrectomy. As ultrasonic shears cannot be used in Japan, we used monopolar curved scissors in the 1st arm, Maryland bipolar forceps in the 2nd arm, and Cadiere forceps in the 3rd arm of the robot. The assistant retracts the stomach or pancreas, operates the stapler, and applies clips. To shorten the console time, the assistant used ultrasonic shears (laparoscopic coagulating shears; LCS) to dissect the omentum and perigastric lymph nodes along the lesser curvature in cases 4–10 except case 9. The stomach was extracted through a 4 cm incision at the upper abdomen, and distal gastrectomy was done. Reconstruction by the Billroth-I method was performed through this incision using a 29 mm circular stapler.

Statistical analysis was performed using the unpaired Student’s t test. P values <0.05 were considered statistically significant.

## Results

**Fig. 2:**
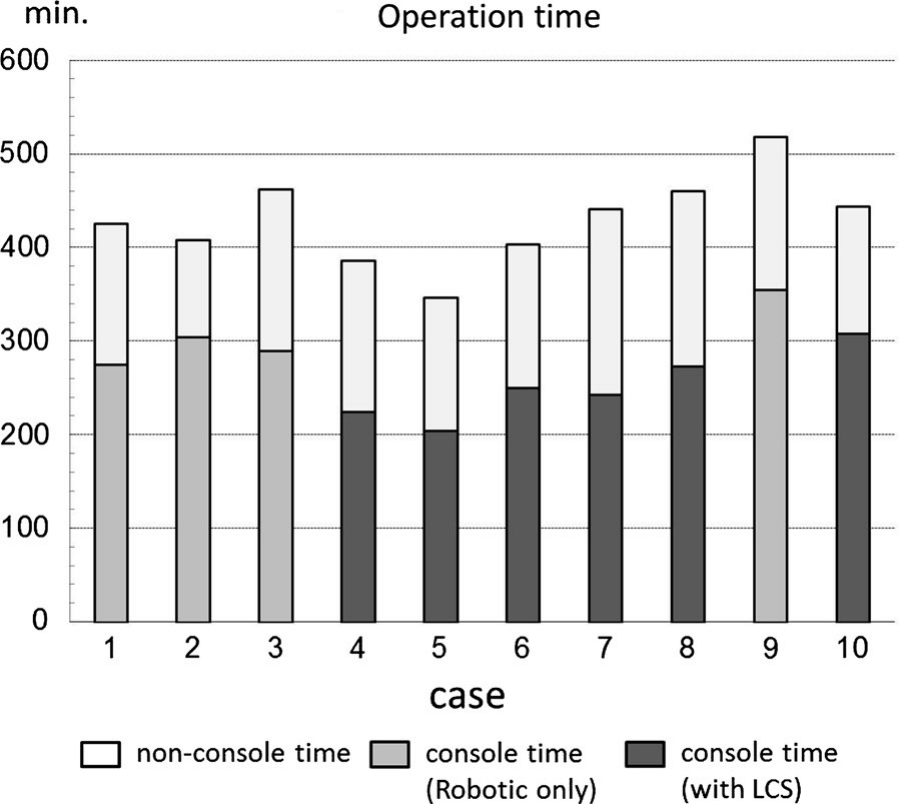
Operation time of each case. The mean console time of 251 min in those with LCS assist was significantly shorter than that of 306 min in cases where only robotic devices were used

Table 1 shows the clinicopathologic characteristics and operative outcomes of the patients. Although the preoperative clinical staging of all the patients was lower than cT1N0M0, one patient (case 3) had a pT2 lesion. The number of dissected lymph nodes was satisfactory to evaluate pathological metastasis pathologically. The mean operative time was 454 min in cases where only robotic devices were used, and 414 min in those with LCS assist. Although the operative time was shorter with LCS assist, there was no statistically significant difference. However, the mean console time of 251 min in those with LCS assist was significantly shorter than that of 306 min in cases where only robotic devices were used (Fig. 2). The difference between these means console time was −55.5 min, 95 % confidence interval −108.9 to −2.1 min; p = 0.0435. The estimated blood loss was small. Postoperative complications included delayed gastric emptying in case 5 and liver dysfunction in case 6, both were slight and transient with short hospital stays.

## Discussion

Robot-assisted laparoscopic surgery provides a 3-dimensional view and articulated movement without physiologic tremor. In general, robotic surgery is reported to require a longer operation time than laparoscopic or open conventional surgery. The prolonged operation time is caused by the additional set-up time for the robotic arms, however, this preparatory time period could be shortened [7]. Another reason for delay time may be the restriction on devices that can be used in Japan. Ultrasonic shears is a useful device to dissect the tissue including vessels. It can securely occlude not only arteries but also veins and lymphatic vessels. We used ultrasonic shears mainly in two operative procedures carried out by the assistant through the assist port. The greater omentum was divided and dissected using the ultrasonic shears toward the lower pole of the spleen. After the clipping of the roots of the left gastroepiploic vessels, the division of the omentum was continued downward to the pylorus. This division of the omentum needs less sophisticated manipulation and is quicker. Another procedure is the dissection of perigastric lymph nodes around the lesser curvature up to the esophagogastric junction. It is sometimes difficult to achieve hemostasis by monopolar or bipolar devices. Application of clips takes time because the clips have to be loaded one by one. The ultrasonic shears are effective in sealing the vessels in this field. Thus, the assistant support using ultrasonic shears was significantly effective in shortening the console time (Fig. 2). In case 9, all dissection procedures were performed by robotics only without ultrasonic shears assistance, however, the console time in this case was 355 min. Although we expect the learning effect to shorten the console time, this trial was not successful. Of course, there remains a limitation of statistical analysis because of the small number of cases, a further analysis with more cases will be recommended.

Noshiro et al. [10] reported that robot-assisted distal gastrectomy using electric cautery instruments without ultrasonic-activated devices was feasible and safe with respect to blood loss, lymph node dissection, and complications. If a variety of devices were available for selection, it would increase the possibilities for performing a sophisticated operation.

Ultrasonic energy instruments or a vessel sealer would be helpful for carrying out effective operative procedures in a shorter time. At the present time, assistant use of ultrasonic shears is useful in robotic gastrectomy.

## Abbreviations

RAG: robot-assisted gastrectomy (RAG)
LCS: laparoscopic coagulating shears
